# Photovoltaic and flexible deep ultraviolet wavelength detector based on novel β-Ga_2_O_3_/muscovite heteroepitaxy

**DOI:** 10.1038/s41598-020-73112-1

**Published:** 2020-09-30

**Authors:** Bhera Ram Tak, Ming-Min Yang, Yu-Hong Lai, Ying-Hao Chu, Marin Alexe, Rajendra Singh

**Affiliations:** 1 Department of Physics, Indian Institute of Technology, Delhi, New Delhi, 110016 India; 2 Department of Physics, University of Warwick, Coventry, CV4 7AL UK; 3 Department of Materials Science and Engineering, National Chiao Tung University, Hsinchu, 30010 Taiwan

**Keywords:** Materials science, Optics and photonics

## Abstract

Flexible and self-powered deep ultraviolet (UV) photodetectors are pivotal for next-generation electronic skins to enrich human life quality. The fabrication of epitaxial β-Ga_2_O_3_ thin films is challenging on flexible substrates due to high-temperature growth requirements. Herein, β-Ga_2_O_3_ ($\stackrel{-}{2}$ 0 1) films are hetero-epitaxially grown on ultra-thin and environment-friendly muscovite mica which is the first time β-Ga_2_O_3_ epitaxy growth on any flexible substrate. Integration of Gallium oxide with muscovite enables high-temperature processing as well as excellent flexibility compared to polymer substrates. Additionally, the metal–semiconductor-metal (MSM) photodetector on β-Ga_2_O_3_ layer shows an ultra-low dark current of 800 fA at zero bias. The photovoltaic peak responsivity of 11.6 µA/W is obtained corresponding to very weak illumination of 75 μW/cm^2^ of 265 nm wavelength. Thermally stimulated current (TSC) measurements are employed to investigate the optically active trap states. Among these traps, trap with an activation energy of 166 meV dominates the persistence photocurrent in the devices. Finally, photovoltaic detectors have shown excellent photocurrent stability under bending induced stress up to 0.32%. Hence, this novel heteroepitaxy opens the new way for flexible deep UV photodetectors.

## Introduction

Flexible electronics with smart materials are playing a key role to meet market demands of future technologies. Internet of things (IoT), wearable electronics, healthcare and electronic skins (e-skins) are major driving application areas for futuristic smart or flexible devices^1,2^. Along with aforementioned research directions, flexible devices like transistors, photodetectors, memristors, displays and sensors are prerequisite for smart electronics^3–10^. Due to these vast application areas, the global flexible electronics market is expected to grow at about 10.5% growth rate till 2026^11^. In the era of robotics, flexible e-skin with various sensors such as optical, pressure, chemical sensors, etc. will necessitate to function it like human’s skin. E-skin with UV photodetectors can enhance the visuals by imaging which can also act as the electronic eye (e-eye) as well as the second skin^12^. Among the wide spectrum of UV radiation, deep UV (< 280 nm) wavelength is most energetic and dangerous for living beings^13^. This wavelength spectrum is almost completely absorbed by the ozone layer. Therefore, photodetectors working in deep UV range possess negligible background signals at the earth's surface. Deep UV photodetectors also important in emerging applications such as space communications, water disinfection, defense, environmental monitoring etc.^14–16^.

The future of flexible electronics also poses various challenges such as providing integrated power sources, temperature harsh fabrication processing for good quality crystalline materials, and also being environment friendly in nature^17,18^. Power consumption is a serious issue to harvest these flexible photodetectors. Usage of power sources with flexible photodetectors makes them bulky and complex when the requirement of many more sensors on the same platform will occur. Specifically, advanced e-skin photodetectors require elimination of power supply requirements^19^. Therefore, photovoltaic detectors are of vital importance for e-skins. In case of photovoltaic operation, photo-generated charge carriers are collected using built-in electric field at the electrode terminals rather than external applied electric field. The built-in electric field in p–n junction develops at the depletion region due to opposite polarity of semiconductor materials as well as band offsets (for heterojunctions)^20^ whereas in Schottky diodes, the built-in electric field exists at the metal–semiconductor junction.

Nowadays, most of the flexible UV photodetectors are fabricated on polymer substrates such as PET, PDMS, and PEN^21–23^. Generally, devices fabricated on epitaxial materials exhibit better performance than amorphous materials. However, the fabrication of epitaxial thin films on flexible substrates is impossible due to the degradation of these substrates at high-temperatures as required for the epitaxial growth. Muscovite mica is a natural substrate that can resolve both the aforementioned challenges of high-temperature processing and environment friendliness^24^. The muscovite has high-temperature stability up to 650 °C. Ultra-thin mica substrate with few nanometer thickness have been reported^25^. The thickness of flexible substrates must be less than 100 μm to function as an e-skin^26^. Therefore, mica is also a strong contender for e-skins. Moreover, muscovite owes superior Young’s modulus, thermal conductivity, and tensile strength than polymer substrates such as PET, PDMS, PEN, etc^24^. Being two-dimensional (2D) and layered substrate, the surface of muscovite is free from the dangling bonds. According to Koma et al., the thin film growth progresses on 2D materials with weak interactions rather than strong chemical bonding between thin film and substrate^27,28^. Thus, the growth method is called as van der Waals epitaxy. Even with large lattice mismatch, van der Waals epitaxy growth mechanism provides the excellent heteroepitaxial growth. Earlier, many high-quality functional oxides such as Fe_2_O_3_, MoO_2_, VO_2_, yttria-stabilized zirconia (YSZ), CoFe_2_O_4_ have been epitaxially grown on muscovite mica. These functional oxides had lattice mismatch with muscovite ranging from 8 to 36% but it didn’t affect the material quality due to van der Waals epitaxy growth^29–33^.

Nowadays, Ga_2_O_3_ and AlGaN are the most widely used wide bandgap semiconductors for deep UV photodetector applications^14,34–41^. Recently, Ga_2_O_3_ has emerged as a better candidate than AlGaN due to its intrinsic deep UV absorption which eliminates the complexity of heavy doping^42^. Only a few research works are performed on the growth of gallium oxide thin films on flexible substrates. Thin films used in these reports were amorphous^23,43^. So far, the epitaxial deposition of β-Ga_2_O_3_ thin films on flexible substrates has been a major challenge. To fill this research gap, self-powered and flexible deep UV photodetectors based on epitaxial β-Ga_2_O_3_ thin films are reported here. The optically active trap states have also been identified, which contribute towards the persistent photocurrent observed in the devices.

## Results and discussion

**Figure 1:**
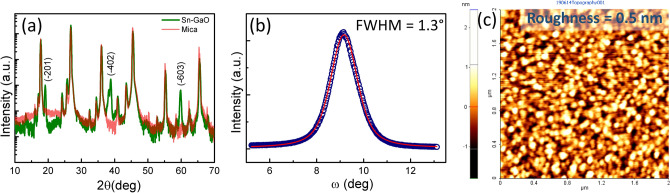
(**a**) XRD 2θ-scan of β-Ga_2_O_3_/muscovite, (**b**) rocking curve of ($\stackrel{-}{2}$ 01)-plane and (**c**) surface morphology of β-Ga_2_O_3_ thin film.

To begin with, structural properties of the Ga_2_O_3_ thin film are investigated by high-resolution X-ray diffraction (HRXRD). Out of plane X-ray 2θ scan of Ga_2_O_3_/muscovite heterostructure is shown in Fig. 1(a). The red color plot depicts the diffraction pattern of muscovite whereas the green color plot represents the diffraction pattern of Ga_2_O_3_/muscovite heterostructure. It shows that ($\stackrel{-}{2}$ 0 1) orientation (JCPDS No.-431012) of monoclinic β-Ga_2_O_3_ was grown on muscovite. Moreover, phi (ϕ)-scan of β-Ga_2_O_3_ ($\stackrel{-}{4}$ 0 1) diffraction is recorded for in-plane structural correlation which is shown in Figure S1. Six peaks are observed in the ϕ-scan of ($\stackrel{-}{4}$ 0 1) plane at the interval of 60°. The ($\stackrel{-}{4}$ 0 1) reflection plane of β-Ga_2_O_3_ possesses two-fold symmetry. Therefore, observation of six peaks in the ϕ-scan represents the three-fold domains in the β-Ga_2_O_3_ epitaxy. Furthermore, the crystalline quality of the thin film is analyzed using the rocking curve measurement of ($\stackrel{-}{2}$ 0 1) plane giving full width at half maximum (FWHM) of 1.3° as depicted in Fig. 1(b). This FWHM of β-Ga_2_O_3_ epitaxy is much lower than the reported MBE grown thin films (1.38–1.99°)^44,45^. HRXRD reveals the epitaxial relationship between β-Ga_2_O_3_ and mica as ($\stackrel{-}{2}$ 0 1) Ga_2_O_3_/(001)Mica. Figure 1(c) shows the surface topography of thin film recorded by atomic force microscopy in 2 × 2 µm^2^ scan area. The root mean square roughness of 0.5 nm is obtained which revealed that the thin film is quite smooth and can be used for device fabrications.

**Figure 2:**
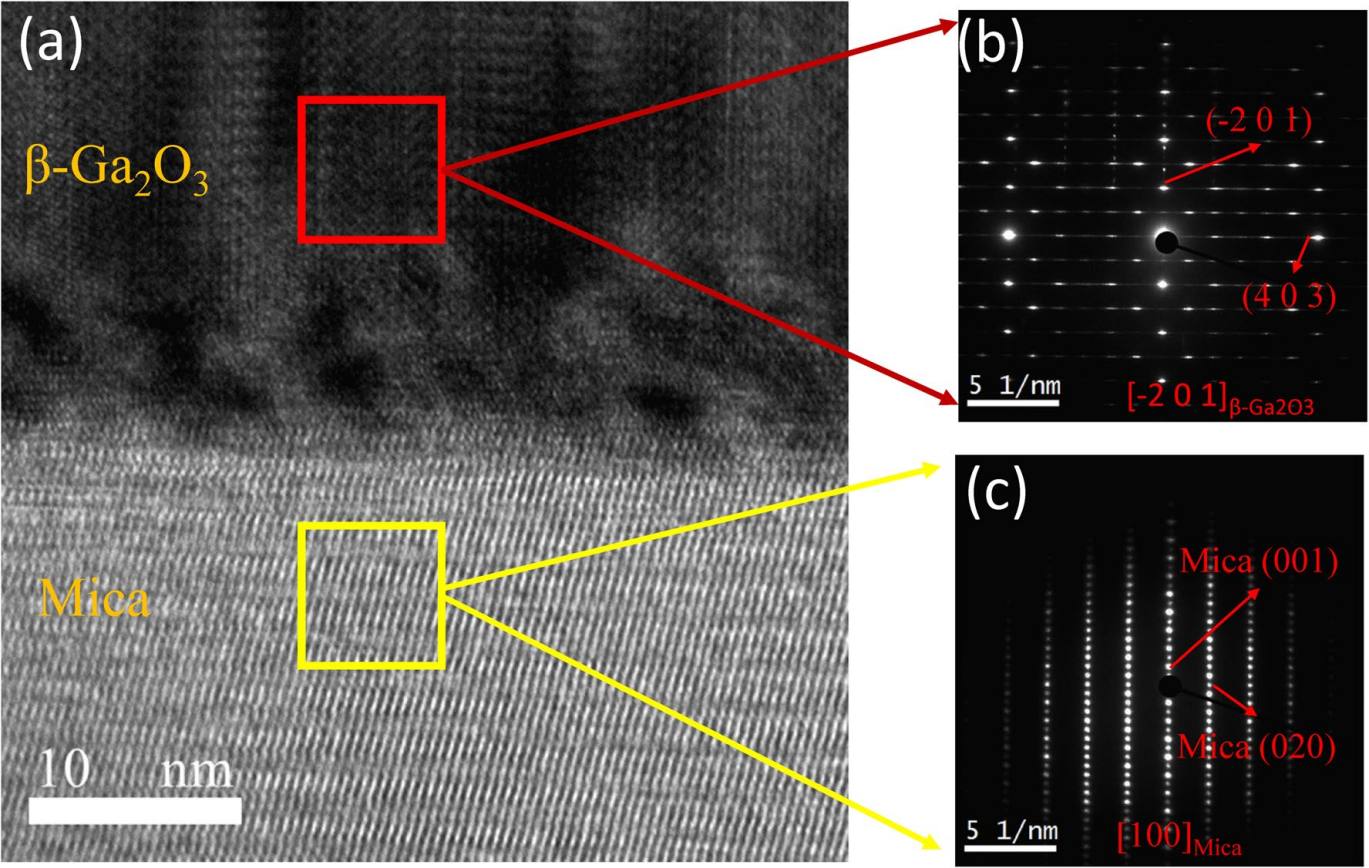
(**a**) Cross-sectional TEM image of β-Ga_2_O_3_/muscovite mica interface, (**b**) SAED pattern of β-Ga_2_O_3_ thin film and (**c**) SAED pattern of muscovite mica.

To further confirm the epitaxial relationship and interfacial characterization, a cross-sectional imaging of the β-Ga_2_O_3_/mica interface is done using a high-resolution transmission microscope (HRTEM) and is shown in Fig. 2(a). The sharp interface between Ga_2_O_3_ thin film and mica is also indicated in Fig. 2(a). The fast Fourier transforms (FFT) of the Ga_2_O_3_ thin film and mica in the reciprocal space are indicated in Fig. 2(b,c) simultaneously. The FFT images also confirm the epitaxial relationship as β-Ga_2_O_3_ ($\stackrel{-}{2}$ 0 1)/mica (0 0 1).

**Figure 3:**
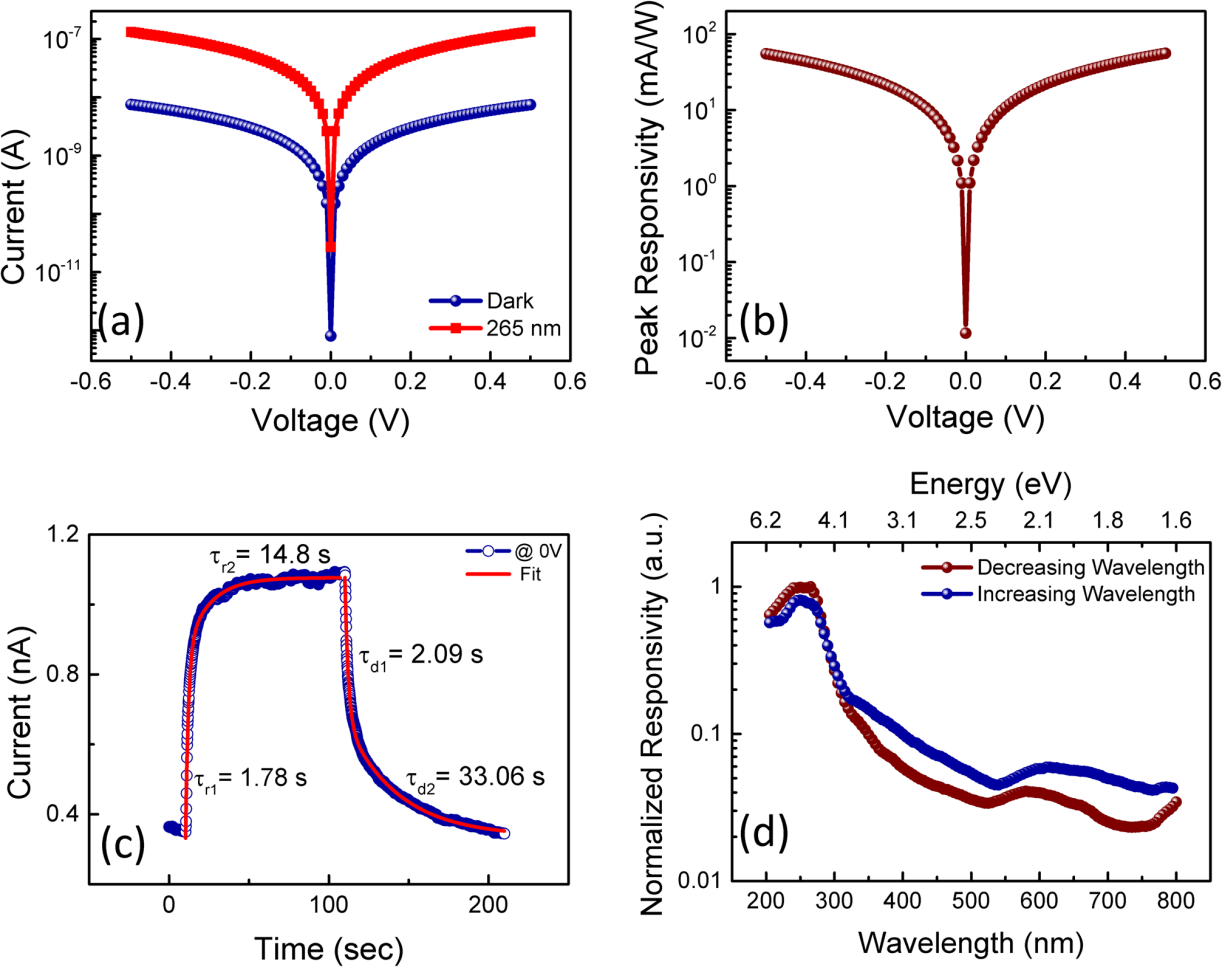
(**a**) Current–voltage measurements under dark and 265 nm wavelength illumination of device, (**b**) peak photoresponsivity, (**c**) time-response measurement at zero bias and (**d**) normalized spectral responsivity.

Further, photoelectrical characterization of the metal–semiconductor–metal photodetector fabricated using heteroepitaxial films is carried out. The current–voltage (I–V) characteristics are measured under dark and 265 nm illumination, as shown in Fig. 3(a). The photocurrent is 45 times larger than the dark current. Here photovoltaic response i.e. zero bias photoresponse is also noticed under illumination, which is known as a self-powered behaviour in photodetectors. This self-powered behaviour is advantageous for the sensors in terms of power consumption which is the need of the current technology. The responsivity of a photodetector is defined as^14,46^

1 $$R= \frac{{I}_{p}-{I}_{d}}{{P}_{\lambda }\times A}$$

where I_d_ and I_p_ denote dark and photocurrent respectively. P_λ_ represents the power density at wavelength λ and A is the effective area of the device under illumination. The voltage-dependent peak responsivity under 265 nm illumination is shown in Fig. 3(b). The zero-bias peak responsivity of 11.6 µA/W was obtained at a weak power density of 75 μW/cm^2^.

For a better performance evaluation of the current work, photovoltaic UV-C detectors are compared in Table 1 with different device architectures. The epitaxial growth of β-Ga_2_O_3_ is also compared due to importance of channel layer in the photodetector performance. Thus, low temperature grown β-Ga_2_O_3_/muscovite heterostructures provided UV-C photovoltaic detector with simple MSM architecture and ultra-low dark current. Flexibility of Ga_2_O_3_/muscovite heterostructure is another important merit over reported photovoltaic UV-C wavelength detectors.

**Table 1:** Comparisons of photovoltaic UV-C detectors with different device structures and channel layer growth methods.

Material structure	Device structure	Growth technique (Ga_2_O_3_)	T_Growth_ (°C)	I_dark_ (A)	I_photo_ (A)	P_opt_ (μW/cm^2^), wavelength	Flexible	Refs.
PEDOT:PSS /Ga_2_O_3_	p–n	MOCVD	860	~ 0.1 p	~ 4 n	1000, 254 nm	No	^55^
PEDOT:PSS /Ga_2_O_3_ nanowire	p–n	CVD	1120	< 0.1 p	–	–, 245 nm	No	^56^
Spiro-MeOTAD/Ga_2_O_3_	p–n	MOCVD	860	75 f.	12 n	80, 254 nm	No	^57^
ZnO/Ga_2_O_3_ microwire	n–n	CVD	1200	~ 1.0 p	~ 5.0 n	1670, 251 nm	No	^58^
SiO_2_/Ga_2_O_3_	MOS	MOCVD	735	~ 2 f.	~ 0.2 n	30, 254 nm	No	^59^
Ni/Ga_2_O_3_	Schottky	Sputtering	750	~ 5 f.	~ 9 p	150, 254 nm	No	^60^
α/β Ga_2_O_3_	Phase Junction	Chemical route	700	1.72 n	211 n	3000, 254 nm	No	^61^
Au/Ga_2_O_3_	MSM	Single crystal	–	0.18 n	2.7 n	1780, 254 nm	No	^62^
Pt/Ga_2_O_3_	MSM	PLD	550	800 f.	36 p	75, 265 nm	Yes	This work

Further, the photovoltaic current obtained under the illumination of 265 nm wavelength is displayed in Fig. 3(c). The photocurrent increases/decreases exponentially with time during switching on/off the illumination. It is observed that photocurrent persisted for several seconds after switching off the illumination. The photocurrent rise and decay curves can be fitted with biexponential Eq. 2 and 3, respectively:^14,23,40^

2 $$I = I_{s} + Ae^{{t/\tau _{{r1}} }} + Be^{{/\tau _{{r2}} }}$$

3 $$I = I_{s} + Ae^{{t/\tau _{{d1}} }} + Be^{{t/\tau _{{d2}} }}$$

where I_s_ represents the steady-state current and A and B are the fitting constants. τ_r1_ and τ_r2_ are fast and slow components of rise times whereas τ_d1_ and τ_d2_ denote the fast and slow components of decay times. Two rise/decay times are obtained by fitting of time response curve using Eqs. (2) and (3). The rise times τ_r1_ and τ_r2_ of 1.78 s and 14.80 s are obtained whereas the decay times of 2.09 s and 33.06 s are acquired. The slow component of rise/decay time is attributed to charge carrier trapping/de-trapping owing to the presence of bandgap states. Larger values of slow components represent the persistent photocurrent in the detector. In this case, high persistent photocurrent is obtained in the time response curve due to the existence of bandgap states in the β-Ga_2_O_3_ films. These trap levels may get charged due to trapping of carriers generated by light.

In order to confirm the existence of defects in the bandgap as well as to identify the peak responsivity, we performed spectral response measurements. The normalized spectral responsivity of the fabricated photodetector is shown in Fig. 3(d). Normalization is carried out by dividing maximum responsivity value to whole spectral responses. The spectral photovoltaic response measurements are performed in the wavelength range of 205 to 800 nm at zero bias. The detector shows the peak photoresponse at 265 nm (4.68 eV) which corresponds to the bandgap of β-Ga_2_O_3_. Thus, the peak photoresponse is observed to lie in the solar-blind region (< 280 nm). Further, the photoresponse at lower wavelengths from 265 to 240 nm remains almost constant and then tends to decrease till 205 nm. In the spectral response curve, various defect bands from 320 to 800 nm are observed. The observation of sub-bandgap absorption is due to optically active recombination centers in β-Ga_2_O_3_ which tend to generate persistent photocurrent.

**Figure 4:**
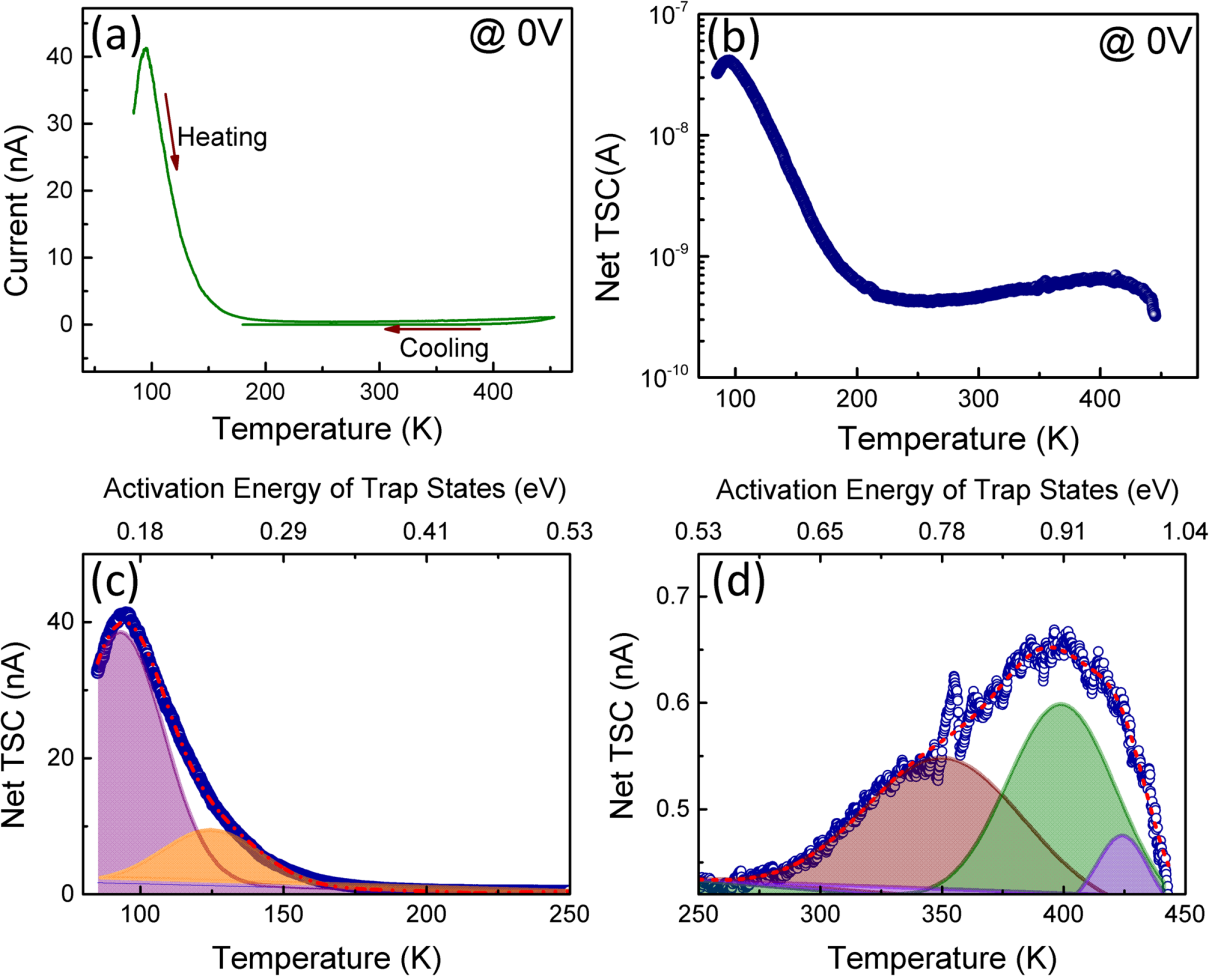
(**a**) TSC curve of the device with 5 K/min heating and cooling rates at zero bias, (**b**) net TSC plot with two broad current peaks (**c**) and (**d**) are the Gaussian fits of both peaks.

Thermally stimulated current (TSC) spectroscopy is employed to explore the role of sub-bandgap states in photocurrent transport. In TSC measurements, all the trap levels are charged optically at a relatively lower temperature to mitigate the thermal emission rate of the carriers from the traps. Thereafter, charge carriers are de-trapped by gradually increasing the temperature at a finite rate under dark conditions and simultaneously recording the stimulated current. Figure 4(a) depicts the thermally stimulated current while heating and cooling at 5 K/min respectively after the charging of trap levels at 85 K. The large difference in the heating and cooling curves is indicative of the presence of traps in the material. The net TSC, which is defined as the total stimulated current minus dark current is shown in Fig. 4(b). In the case of thermally activated electron/hole emission, a particular filled trap starts to emit the charge carriers at a characteristic temperature and later on the emission rate continuously decreases with removal of electron/hole from the trap, generating in this way a current peak. In our particular case, two broad current peaks near about 95 and 400 K have appeared in the net TSC spectra. Both the peaks are fitted with Gaussian to deconvolute the trap states as shown in Fig. 4(c,d) corresponding to peak at 95 and 400 K simultaneously. The first peak is deconvoluted in two peaks centered at 93.3 and 125 K whereas the second peak is deconvoluted into three peaks situated at 351.3, 399.7 and 424.7 K. In the variable heating method of TSC, the activation energy (E_T_) of a trap state at a heating rate of γ can be defined as^47^

4 $${E}_{T}= {kT}_{m}ln\left[\frac{{T}_{m}^{4}}{\gamma }\right]+ {kT}_{m}ln\left(\frac{{N}_{c}{v}_{th}{k\sigma }_{n}}{{E}_{T}}\right)$$

where T_m_ is the characteristic temperature of TSC peak, ${v}_{th}$ is the thermal velocity, N_c_ is the density of states in the conduction band, σ_n_ is the capture cross-section of the trap and k is the Boltzmann constant. The second term in Eq. (4) is very small relative to the first term and hence can be neglected^47^. Therefore, Eq. (4) can be approximated for the activation energy of trap state as^47^

5 $${E}_{T}= {kT}_{m}ln\left[\frac{{T}_{m}^{4}}{\gamma }\right]$$

The activation energies of all the traps corresponding to the aforementioned characteristic temperatures are calculated using Eq. (5). The trap states denoted as E_1_, E_2_, E_3_, E_4_ and E_5_ correspond to the characteristic temperatures of 93.3, 125, 351.3, 399.7 and 424.7 K respectively. The activation energies of all these traps are tabulated in Table 2. These traps have activation energies ranging from 0.1 to 1 eV.

**Table 2:** Distribution of bandgap states calculated from thermally stimulated current spectroscopy.

Traps	Temperature maxima of TSC peak (K)	Activation energy of traps (meV)
E_1_	93.3	166
E_2_	125.0	234
E_3_	351.3	785
E_4_	399.7	911
E_5_	424.7	977

It is observed that E_1_ trap having activation energy of 166 meV is the dominant trap among all the five traps. The activation energies corresponding to other traps E_2_, E_3_, E_4_, and E_5_ are obtained as 234, 785, 911 and 977 meV respectively. Wang et al. have also investigated the traps in β-Ga_2_O_3_ thin films which were grown under similar conditions to current work. Traps E_3_, E_4_ and E_5_ exhibited almost same activation energies as reported in the literature^47^. Other than aforementioned traps, Wang et al. also reported the traps with activation energies 0.43, 0.49, 0.52 and 0.54 eV. However, these traps are not observed in the present work which indicative of better crystalline quality of thin film. The energy of E_1_ and E_2_ levels are relatively shallow and Sn can be the possible origin of these traps. The double donor behavior of Sn is possible due to two different substitutional Ga sites (tetrahedral and octahedral). The behavior of Ge as a double donor with activation energies of 0.18 and 0.21 eV has also been reported using deep level transient spectroscopy (DLTS)^48^. However, further detailed analysis is needed to study the double donor behavior of Ge and Sn in Ga_2_O_3_. Although both dopants have also been reported as a shallow donor with activation energies less than 50 meV^49^. However, a trap with activation energy of 165 meV was also reported in metal oxide vapor phase epitaxy (MOVPE) grown Ga_2_O_3_ thin films using deep level noise spectroscopy^50^. The possible origin of this trap is still unknown. Tadjer et al. reported a trap located 0.23 eV below conduction band in halide vapor phase epitaxial Ga_2_O_3_ which was co-doped by Si and N impurities^51^. Therefore, the E_2_ trap of 234 meV may be originated from unintentional nitrogen doping. The E_3_ trap having activation energy 785 meV is the most common trap center in Ga_2_O_3_. It is reported that Fe doping is responsible for this trap^52^. Remaining E_4_ and E_5_ traps are also common to Ga_2_O_3_. Irmscher et al. reported that Co impurity may be the origin of these deep traps^48^. On the basis of TSC results, the persistence photocurrent of Ga_2_O_3_ self-powered photodetectors is due to five trap levels with activation energies ranging from 0.16 to 0.97 eV. Among them, the E_1_ trap with activation energy of 166 meV had the dominant contribution in persistence photocurrent.

**Figure 5:**
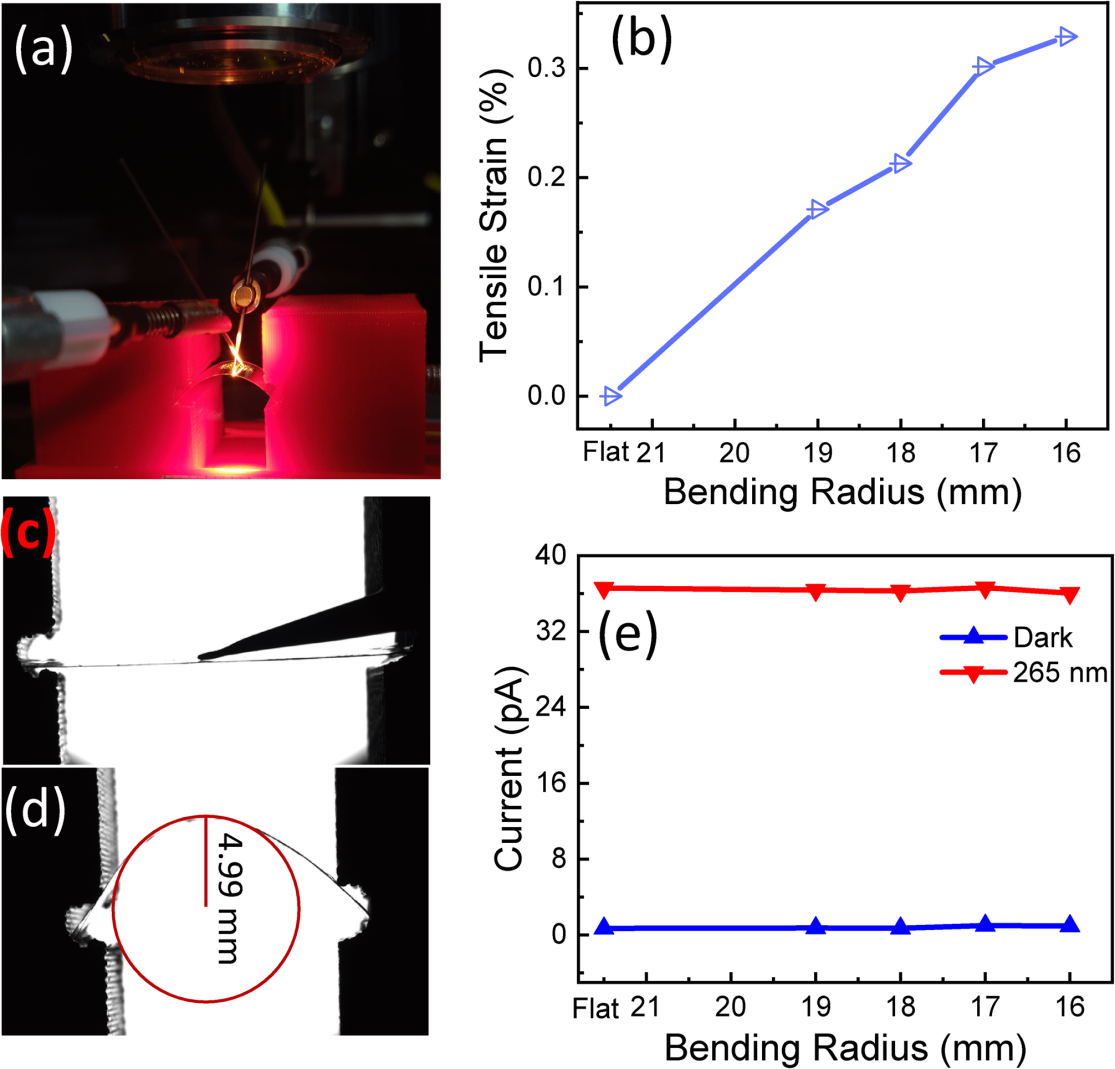
(**a**) Measurement setup for bending tests of the device, (**b**) Tensile strain versus bending radius of the device, (**c**) CCD camera images of a flexible device under flat, (**d**) 16 mm bending radius condition, (**e**) dark and photocurrent of the device at zero bias with bending radius of muscovite.

Further, flexibility tests of photodetectors are performed using the measurement setup shown in Fig. 5(a). The variation of bending radius of the muscovite substrate having 21 mm length was executed from flat condition to 16 mm radius. Figure 5(b) represents the tensile strain in the film with respect to the bending radius. The strain ɛ induced in the film consisting of thickness t_f_ can be determined as^53^

6 $$\varepsilon = \left(\frac{{t}_{f}+{t}_{s}}{2{R}_{c}}\right)\left(\frac{1+2\eta +\chi {\eta }^{2}}{\left(1+\eta \right)\left(1+\chi \eta \right)}\right)$$

where t_s_ is the thickness of the substrate, η is defined as t_f_/t_s_, R_c_ is the radius of the curvature due to bending and χ is denoted as Y_f_/Y_s_, Y_f_ and Y_s_ are Young’s modulus of thin film and substrate respectively. The value of Young’s modulus of Ga_2_O_3_ and muscovite are 261 and 190 GPa, respectively^24,54^. The maximum strain of 0.32% is induced in the film for photoelectrical measurements. Both photocurrent and dark current are recorded with varying bending radii which are shown in Fig. 5(e). The CCD camera images of the mica sheet is also recorded corresponding to flat and bending conditions which are shown in the inset of Fig. 5(c,d). The radius of curvature was 4.99 mm analogous to a 16 mm bending radius. The photocurrent and dark current showed no obvious change and remained almost constant under bending tests which is desirable feature for a flexible photodetector. The excellent performance under bending may be attributed to good structural stability towards mechanical strain of thin film. Other than the structural stability, durability of extremely good metal–semiconductor contacts with mechanical strain resulted in stable dark and photocurrent. In the earlier article, amorphous gallium oxide/muscovite-based photodetectors are reported to possess about one order enhancement in dark as well as photocurrent^7^. Table 3 shows the comparisons of flexible UV-C photodetectors based on gallium oxide thin films. Hence, epitaxial Ga_2_O_3_/muscovite photodetectors demonstrate much better performance stability towards mechanical flexibility and also has zero power consumption which are very useful properties for e-skins.

**Table 3:** Comparisons of self-powered and flexible UV-C photodetectors based on Ga_2_O_3_.

Heterostructure	Dark current	Photocurrent	Photovoltaic	Bending stability	Refs.
a-GaO/PEN	~ 10^–13^ A @10 V	~ 10^–8^ A	No	No	^23^
a-GaO/PET	~ 10^–9^ A @5 V	~ 10^–10^ A	No	No	^43^
a-GaO/Muscovite	~ 10^–6^ A @5 V	~ 10^–5^ A	No	No	^7^
β-GaO/Muscovite	8 × 10^–13^ A @0 V	3.6 × 10^–11^ A	Yes	Yes	This work

## Conclusions

Heteroepitaxy growth of β-Ga_2_O_3_($\stackrel{-}{2}$ 0 1) on layered muscovite mica substrate has been carried out using pulsed laser deposition. The epitaxial relationship between β-Ga_2_O_3_ and muscovite is also established using HRXRD and HRTEM. The MSM photodetector made on Ga_2_O_3_ thin film shows a photovoltaic response corresponding to 265 nm illumination. It is noticed that the photoresponse is influenced by persistence photocurrent. Thermally stimulated current spectroscopy is employed to investigate trap levels in the devices. The trap with an activation energy of 166 meV is found to dominate the persistence photocurrent among five different trap levels. In addition, the result of bending tests demonstrates excellent stability in photocurrent at zero bias. The obtained results provide a new perspective for photovoltaic and flexible deep UV photodetectors on epitaxial β-Ga_2_O_3_.

## Experimental section

### Sample fabrication

The heteroepitaxy of β-Ga_2_O_3_ on cleaved muscovite (001) is grown using a pulsed laser deposition system (Nobert’s electron beam and laser deposition equipment). A KrF excimer laser having 248 nm wavelength is utilized to ablate the tin-doped (1 wt%) gallium oxide target. The laser fluence is kept at 1.5 J/cm^2^ with a repetition rate of 5 Hz. The muscovite substrate is placed at a 50 mm distance from the gallium oxide target. The temperature of 550 °C is maintained during the deposition at an oxygen pressure of 5 × 10^–3^ mbar.

### Material characterizations

The X-ray diffraction is acquired with Cu K_α_ (λ = 1.54 Å) source using a Panalytical Xpert Pro system. The topography of the thin film is mapped using XE-100 Park AFM system. Nano-structural characterization is performed using field emission transmission electron microscope (FE-TEM) system (Jeol, JEM-F200) with a point resolution of about 0.23 nm.

### Device fabrication and characterization

The 600 µm long interdigitated electrodes (IDE) at 200 µm spacing are patterned using a metal mask. E-beam evaporation system (Scientific Vacuum System Ltd.) is used to fabricate Pt (20 nm)/Au (80 nm) metal contacts for IDE. Photoelectrical measurements are carried out in an optical cryostat (Janis VPF-700). For Spectral photoresponse distribution, the wavelength of incident light is tuned using a monochromator and Xenon lamp. At the same time, the output current is measured using an electrometer (Keithley, 6517). Further, the photocurrent spectrum is calibrated by power spectrum of the Xenon lamp for photoresponse measurement. The photoresponse distribution is normalized with respect to the maximum response value.

### Thermally stimulated current measurements

For the TSC experiment, the device is cooled down from room temperature to 85 K under the dark conditions in a cryostat. The trap filling in Ga_2_O_3_ is performed under 265 nm wavelength illumination at 85 K. To ensure the better trap filling, the device is illuminated at zero bias for 20 min with 20 μW/cm^2^ power density of 265 nm light. Further, illumination is turned off and charge carriers were de-trapped by heating the device up to 450 K with a constant heating rate of 5 K/min. Simultaneously, the thermally stimulated current is recorded with increasing temperature. The dark current measurements are also conducted with temperature to ensure the net TSC in the device.

## Supplementary information

Supplementary information

## Data Availability

The raw/processed data required to reproduce these findings cannot be shared at this time as the data also forms part of an ongoing study. In future, it will be provided on request by corresponding author.
