# Supplementary material for: Photovoltaic and flexible deep ultraviolet wavelength detector based on novel β-Ga2O3/muscovite heteroepitaxy
Source: Sci Rep. 2020 Sep 30;10:16098. doi: 10.1038/s41598-020-73112-1 (PMC7528161; doi:10.1038/s41598-020-73112-1)
Supplement: Supplementary file 1 — Supplementary information [file 41598_2020_73112_MOESM1_ESM.docx]

Supporting Information

Photovoltaic and Flexible Electronic Skin for Deep Ultraviolet Wavelength Detection Based on Novel β-Ga_2_O_3_/Muscovite Heteroepitaxy

Bhera Ram Tak,^1,*^ Ming-Min Yang,^2^ Yu-Hong Lai,^3^ Ying-Hao Chu,^3^ Marin Alexe^2^ and Rajendra Singh^1^

^1^Department of Physics, Indian Institute of Technology Delhi, New Delhi-110016, India

^2^Department of Physics, University of Warwick, Coventry-CV4 7AL, UK

^3^Department of Materials Science and Engineering, National Chiao Tung University, Hsinchu 30010, Taiwan





Figure S1 Phi-scan of (-401) plane of β-Ga_2_O_3_ thin film
